# The Association between Delirium and In-Hospital Falls: A Cross-Sectional Analysis of a Delirium Screening Program

**DOI:** 10.1155/2023/1562773

**Published:** 2023-01-30

**Authors:** Benjamin Kalivas, Jingwen Zhang, Kristine Harper, Meghan K. Thomas, Jennifer Dulin, Justin Marsden, Patrick Robbins, Kelly J. Hunt, Patrick D. Mauldin, William P. Moran, James Rudolph, Marc Heincelman

**Affiliations:** ^1^Department of Medicine, Medical University of South Carolina, 135 Rutledge Avenue, MSC 591, Charleston, SC 29425, USA; ^2^Department of Psychiatry and Behavioral Sciences, Medical University of South Carolina, 67 President Street, MSC 862, Charleston, SC 29425, USA; ^3^Quality Department, Medical University of South Carolina, 150 Ashley Avenue, MSC 585, Charleston, SC 29425, USA; ^4^Department of Internal Medicine, University of South Florida, 12901 Bruce B. Downs Blvd, Tampa, FL 33612, USA; ^5^Department of Psychiatry and Behavioral Sciences, Emory University School of Medicine, 12 Executive Park Drive NE, Atlanta, GA 30329, USA; ^6^Department of Public Health, Medical University of South Carolina, 135 Cannon Street, Ste 303 MSC 835, Charleston, SC 29425, USA; ^7^Brown University Department of Medicine, 593 Eddy Street, Providence, RI 02903, USA

## Abstract

**Methods:**

A cross-sectional study using delirium screening and falls reports was used to measure the association between delirium and falls. All inpatient data from August, 2018, to January, 2020, at a large academic medical center were analyzed. A multivariable logistic regression of 29,655 hospital admissions was used to understand the association between in-hospital delirium and falls.

**Results:**

Analysis revealed a delirium rate of 12.5% (*n* = 3,707) of all admissions and 286 (0.9%) admissions with falls; of the falls studied, 37.6% of these patients screened positive for delirium during their admission. Relative to those who screened negative for delirium, admissions that screened positive for delirium had a 2.81 increased odds of falling.

**Conclusions:**

Delirium and falls are related. This strong association should motivate health systems to look closely at both problems. Falls and delirium can both have immense impacts on the patient and the health system. The powerful association between them provides a window to reduce these additional patient harms. More specifically, a modern delirium screening tool should be used as part of routine risk assessment focused on reducing in-hospital falls.

## 1. Introduction

In-hospital falls are considered complications of hospitalization, affecting both the patient and the health system. The World Health Organization defines a fall as an “event that results in a person coming to rest inadvertently on the ground, floor, or other lower level” [1]. Falls with resultant hip fractures are often sentinel events, included in the Center for Medicaid and Medicare Services' (CMS) Patient Safety and Adverse Event Composite, used to objectively evaluate adverse events in a hospital [2]. Falls are the most commonly reported safety event, occurring approximately 3.5 times per 1000 patient days [3], and are particularly common and harmful in older adults [4]. Many patient-level factors contribute to falls including arthritis; depressive symptoms; orthostasis; impaired vision, balance, gait, or muscle strength; polypharmacy; and impaired cognition [5].

Delirium is an acute neuropsychiatric disturbance caused by acute medical illness, defined as disruption in attention and acute change in alertness and cognition [6, 7]. It is difficult to diagnose and prevent [8, 9], and greatly increases a patient's morbidity and mortality, resulting in a substantial financial impact [6, 10]. Common risk factors for delirium overlap considerably with risk factors for falls, including advanced age, sensory impairment, cognitive impairment, and dementia [11, 12].

Delirium and falls not only share risk factors but also the associated negative outcomes. Both negatively impact mortality, length of stay, risk of discharge to a higher level of care, and increased cost of hospitalization [6, 13–16].

The disruption of cognition as a risk factor for falls can be further described as chronic, as in dementia, or acute, as in delirium. Delirium is an important and potentially modifiable risk factor for falls [12, 17]. Furthermore, preventing delirium can potentially reduce the associated morbidity, such as falls. A meta-analysis that included 4 studies with 1038 patients and looked at falls as an outcome of delirium-prevention interventions showed a 62% reduction in falls [17].

A challenge that remains is unifying cognitive assessment and fall risk. Two widely used fall scales, the Morse Fall Scale and the Hendrich II Fall Risk Model, both score cognitive impairment and mental status change in assessing fall risk [18, 19]. However, both require subjective interpretation of the deficit. A patient's mental status, particularly in older patients, can fluctuate with acute illness. Delirium is an acute change in cognition, and thus likely an important risk factor for falls. Despite this, delirium specifically is often absent in fall risk tools [12]. Essential to prevention is the identification of patients at the highest risk of falling. Although numerous fall scales have been validated in different patient populations [18–20], each is imperfect and has advantages and disadvantages in certain populations and care settings [18, 21–23].

The goal of the study is to use the data from a hospital-wide delirium screening program at a large, tertiary care, academic hospital to strengthen the known association between delirium and falls and emphasize the practicality and importance of using accessible, bed-side delirium screening tools.

## 2. Methods

This study is a cross-sectional study of hospitalized patients at a 740-bed, tertiary care, academic hospital. Medical University of South Carolina (MUSC) Institutional Review Board (IRB) approval was obtained, and informed consent was waived. The reporting for this analysis follows the STROBE reporting guidelines.

### 2.1. Study Population and Data Collection

All data were obtained from the MUSC Data Warehouse. The data set included all adult inpatient encounters from August, 2018, to January, 2020. Falls data were obtained from a hospital incident reporting database that records all falls that occur in the hospital for reporting to the Center for Medicare and Medicaid Services (CMS) and the Joint Commission.

**Figure 1:**
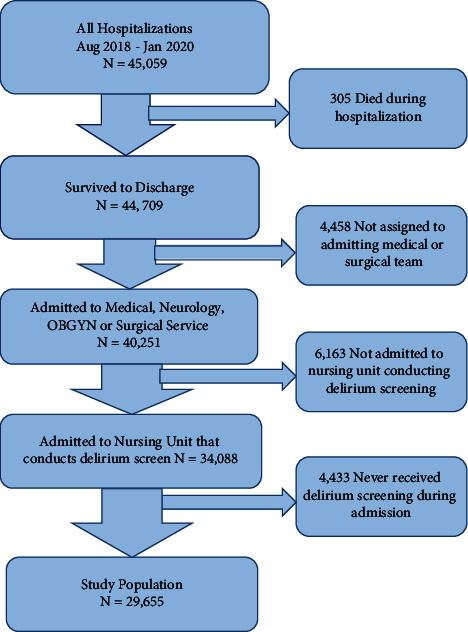
Consort diagram: study population and exclusions.

Inclusion criteria were any adult (18 years and older) admitted to our hospital during this time period. Inpatient admission was defined as an admission to one of the inpatient services, including all medical, surgical, neurologic, and OBGYN services, and excluding radiology, laboratory, and procedural areas that would not have been admitted to an inpatient service. Any patient who did not receive a delirium screen during hospitalization, was not admitted to a prespecified service, or died during the hospitalization was excluded (Figure 1).

### 2.2. Delirium Screening

Starting in 2017, MUSC sought to improve patient care and reduce falls by enhancing the ability to detect delirium in non-ICU patients. The program included twice-daily nursing administration of the Brief confusion assessment method (bCAM). The bCAM is a validated delirium screening tool that assesses level of arousal, attention, and presence of disorganized thinking [24]. The delirium screening initiative was expanded over an 18-month period and implemented in phases to include all adult in-patients. By August 2018, all adults admitted to the hospital were screened for delirium at least twice-daily, using the bCAM in the non-ICU setting and the CAM-ICU in the ICU setting. Results are recorded as positive or negative in the electronic medical record (EMR). Eighty-seven percent of all patients received a CAM-ICU or bCAM screen at least once during their hospitalization. Since full implementation, there has been 63% compliance with the tool on a per-opportunity-to-screen basis across all non-ICU adults during the time frame of this study. The CAM-ICU is administered on every ICU patient at a minimum of every 12 hours. Across all ICUs, compliance with the screening tool was approximately 88% during the timeframe of this study. Results for both ICU and non-ICU were recorded in the same location in the EMR, and the results are viewed the same in data extraction. If screening was not completed, it was not recorded and treated as missing. If the patient never received a screening during the admission, they were excluded from the analysis.

If a patient screens positive for delirium, nursing would initiate the “Acute Confusion Care Plan” which included a series of nonpharmacologic, safety interventions aimed at reducing harm. The care plan was a one-time intervention that provided reminders for safety measures such as bed alarms, call-bells within reach, a reduction in unnecessary stimuli, and calendar and clock access.

Patients were defined as positive for delirium (bCAM or CAM-ICU positive) with any positive delirium screen (bCAM or CAM-ICU) during their hospitalization, and they were defined as negative for delirium (bCAM or CAM-ICU negative) if all screens during the hospitalization were negative.

### 2.3. Fall Records

Falls were collected from the hospital's centralized documentation of adverse outcomes, maintained by the Department of Quality and Safety for the purpose of quality improvement. This monitoring system defines a fall as an event in which there is uncontrolled, downward displacement of a patient's body from a standing, sitting, or lying position. This database includes both preventable and unpreventable falls regardless of whether an injury is incurred. This data set also includes any Morse Fall Risk Score performed, used institutionally as the fall risk scoring tool. The Morse is completed by nursing at the time of admission, every shift and any change in clinical condition. Patients who scored as “high fall risk,” based on the Morse were treated as “high-risk” and appropriate fall-prevention bundles were put in place. This protocol did not change during the duration of the study period. This data set was then integrated into the larger data set by matching medical record number and hospitalization data.

### 2.4. Outcome Definition and Covariates

The primary outcome was an in-hospital fall. The main independent variable was delirium, measured through CAM-ICU and bCAM screen results. Covariates included demographics such as age, gender, race, and marital status; distance from our hospital; history of alcohol abuse; Charlson comorbidities using hospitalization encounter ICD-10 diagnoses codes; medications ordered; and discharging physician's specialty service. Poverty as a covariate was defined as a dichotomous variable assigned a value of 1 if the patient's zip code had ≥25% of citizens below the federal poverty level (FPL) using the patient zip codes linked to 2010 Census data.

### 2.5. Statistical Analysis

Univariate analyses were performed between patients with positive and negative delirium screens using Pearson's *χ*^2^ for categorical variables and the Student's *t*-test for continuous variables. A multivariable logistic regression model was used to fit a predictive model for the outcome of the fall. To account for the low event number of falls, the Firth method was used to determine significance [25, 26]. Given the large number of variables, a multivariable analysis using backwards selection with a *p* value cutoff of <0.2 was performed to identify the most parsimonious model. Collinearity was assessed and when two variables exhibited high correlation, one was dropped from the model based on clinical relevance. All statistical analyses were performed using SAS 9.4 (SAS Institute Inc., Cary, NC) and significance was determined at the 5% level.

## 3. Results

There were 29,655 patient admissions included in the analysis (Figure 1) and 3,707 (12.5%) patients screened positive for delirium at least once during their hospitalization. Falls occurred in 2.8% (105/3,707) patients with delirium compared to 0.7% (181/25,948) patients without delirium.

The demographic profile comparison of delirious and nondelirious patients is shown in Table 1. Patients who screened positive for delirium during the hospitalization were significantly older (median age of 65) compared to the nondelirious group (median age of 60), (*p* value <0.001). Patients who screened positive for delirium were significantly more likely to have a history of alcohol abuse, dementia, myocardial infarction, cerebral vascular disease, congestive heart failure, peptic ulcer disease, cancer, and liver disease. The individual Charlson comorbidity diagnosis that were included in the logistic regression are displayed in Table 1. Patients who screened positive for delirium were significantly less likely to be female than nondelirious patients (48.0% versus 51.2%, respectively). Patients who screened positive for delirium were significantly more likely to be in the ICU at some point during their admission (62.8%) than nondelirious patients. A diagnosis of dementia was significantly more common in the delirious group (14.9%) compared to the nondelirious group (3.1%). Patients who screened positive for delirium were significantly more likely to have orders for benzodiazepines (61.9%) and antipsychotics (51.3%); however, nondelirious patients were significantly more likely to have orders for opioids (82.9%) and anticholinergic medications (64.4%).

Table 2 describes the distribution of falls among our population and characteristics of those who fell. There were 300 falls included in the study, which occurred over 286 hospitalizations, representing 0.96% of the total analyzed admissions. There were 284 unique patients who fell, with some patients recording multiple falls during their hospitalization and 2 patients falling during two separate hospitalizations. Patients who fell had a median age of 61 and 42.7% of those who fell were female. Fall risk scoring was completed in 86.3% of patients prior to their fall. Of these patients, 74.5% were considered “high-fall risk” with a Morse Fall Score of greater than 50. The mean Morse Fall Score was 68.9 ± 23.0. Among patients who fell, 15.7% sustained an injury. The majority of patients (95.1%) who fell only had one fall. A smaller portion (4.2%) had 2 falls and even smaller number (0.7%) had more than two falls.

Of the 286 hospitalizations containing at least one fall, 105 (36.7%) screened positive for delirium during the admission. The multivariable logistic regression for odds of in-hospital fall (Table 3) shows that delirious patients had 2.81 the odds of falling compared to nondelirious patients when controlling for the other clinical variables (OR = 2.81 (95% CI: 3.70)). The regression demonstrates that admissions that were eventually discharged from the ICU (OR = 3.64 (95% CI: 0.97, 9.82)) or the OBGYN service (OR = 2.82 (95% CI: 1.475, 5.036)) were the only clinical variables that had higher odds ratios associated with falls than delirium; however, these were much lower in number than the other groups. Benzodiazepine order was also strongly associated with falls with an odds ratio of 2.03 (95% CI: 1.56, 2.66).

## 4. Discussion

Our analysis of a large data set of hospitalized patients revealed a strong association between the occurrence of delirium and an in-hospital fall, with an odds ratio of 2.81. This association is not a new finding; however, most studies look at cohorts that are already at high risk for either delirium or falls, or both, in contrast to our study which included all adult admissions [12]. These results add to the known association by the use of multivariable analysis that controls for important demographics as well as clinical variables that can contribute to both delirium and falls. ICU admission was the only variable that was a stronger predictor of falls than delirium. Importantly, ICU admission is also a strong predictor of delirium [27].

Delirium screening should be a piece of high-quality care of all hospitalized adult patients. It is already a guideline-driven standard of care in the ICU [28]. This analysis confirms and strengthens the known association between delirium and falls by using a practical and easily adoptable delirium screening tool, the bCAM. This delirium tool was integrated into the assessment of all patients and has the potential to identify patients at risk for falls as well as other well-described negative outcomes associated with delirium. There is no test for delirium that is both practical for wide-spread use and perfectly accurate [29]. The use of the bCAM in this study may further strengthen its results because of its modest sensitivity (78%) and high specificity (97%) which would bias towards the null and under-estimate delirium [24].

Furthermore, the addition of delirium screening in the non-ICU setting enhances the care team's ability to more accurately understand a patient's mental status. Delirium is often under-recognized, with some studies suggesting a diagnosis in less than a third of cases [8, 30]. The hypoactive subtype which has a more subtle clinical presentation, carries an equivalent impact on morbidity and mortality and can be easily overlooked [31]. Identifying a change in mental status is an essential first step in reducing negative outcomes associated with delirium, including falls [13, 14]. Effectively preventing delirium cannot be done if we are not measuring it in real time. Prevention protocols can be effective and have been shown to reduce not only the incidence of delirium but also falls, length of stay, and hospital readmission [17, 32].

The strong association between delirium and falls suggests that a greater consideration of delirium should be included in fall risk assessment. Although the commonly used fall risk assessments acknowledge mental status abnormalities as part of their scoring, they do not concurrently diagnose delirium [18, 19]. Furthermore, the fall risk assessments yield a very low specificity and could be enhanced with more accurate mental status assessment [33, 34].

Clinically, adding this information may create an opportunity for reducing a patient's risk of falling. Delirium can be managed by diagnosing and treating the new or already known underlying medical illness in combination with other behavioral and environmental interventions [7, 35]. It is challenging to reduce a patient's risk of falling. Systematic reviews of multifactorial, patient-centric approaches have shown anywhere from no change to a reduction in falls [23, 36]. Considering the association between falls and delirium, by detecting and managing delirium effectively, there is a great opportunity to reduce falls [17, 37].

The study has several limitations. It is a single-site study which may limit generalizability, and despite hospital-wide efforts, one third of admissions were not included in the analysis for various reasons (Figure 1). Additionally, the incidence of falls is relatively low (1%). Although a strong correlation is demonstrated and supported with statistical analysis, the methodology is ultimately a retrospective study and thus cannot prove a causative relationship between delirium and falls. Furthermore, the timing of falls during the admission was not compared to the timing of positive delirium screening, limiting the association to the entire admission, not the specific events. Due to changes in staffing and work flow from the SARS-COV-2 pandemic, compliance with delirium screening was greatly reduced. Because of this, further data collection was postponed after January 2020.

The broad inclusion and limited exclusion criteria stress the importance of the results across diverse patient populations. Further studies could evaluate the impact of age, diagnosis of admission, and whether or not the fall resulted in injury. Additional analysis looking at motor subtypes of delirium would be an area of future study. Use of the Richmond Agitation Sedation Scale (RASS) as a means of qualifying hyperactive or hypoactive delirium would be an interesting future area of investigation.

In summary, the development of delirium is strongly correlated with a patient experiencing a fall during a hospitalization. Institutions may consider integrating fall-prevention and delirium-prevention tools, such as regular screening, to identify and better manage these interrelated hospital complications.

## Figures and Tables

**Table 1:** Demographics of delirious and nondelirious patients.

	*bCAM or CAM-ICU*	
	Negative (*n* = 25,948)	Positive (*n* = 3,707)
Outcome: fall (%)	0.7	2.8
Age (mean, SD)	57.3 ± 17.2	63.1 ± 16.6
Age (median)	60.0	65.0
*Age group (%)*		
<50	30.6	19.3
50–64	30.5	28.6
65–79	31.3	37.5
80+	7.7	14.6
*Gender (%)*		
Male	48.8	52.0
Female	51.2	48.0
*Race (%)*		
Black	34.9	38.4
Other	3.5	3.2
White	61.6	58.4
*Marital status (%)*		
Married	48.8	43.0
Other	18.8	24.4
Single	32.5	32.6
Distance (mean, SD)	65.4 ± 127	62.6 ± 150^*∗*^
Distance (median)	39.6	28.6
Far to MUSC (distance >50 miles)	46.2	44.2
Poverty (%)	29.6	31.6
Score of CCI (mean, SD)	4.0 ± 3.2	5.1 ± 3.1
Score of CCI (median)	3.0	5.0
*Score of CCI (%)*		
0	12.8	5.3
1-2	25.6	16.2
3-4	25.3	25.9
5+	36.3	52.6
*Medication exposure (%)*		
Antipsychotic	27.5	51.3
Anticholinergics	64.4	55.3
Opioid	82.9	78.6
Benzo	37.7	61.9
ICU during hospitalization (%)	15.3	62.8
Alcohol abuse (%)	6.2	14.0
Dementia (%)	3.1	14.9
Myocardial infarction (%)	11.9	18.1
Congestive heart failure (%)	18.7	27.1
Cerebrovascular disease (%)	8.6	26.5
Chronic pulmonary disease (%)	20.4	22.9
Rheumatoid arthritis (%)	5.1	5.8^*∗*^
Peptic ulcer disease (%)	1.6	2.5
Uncomplicated diabetes (%)	10.9	10.6^*∗*^
Complicated diabetes (%)	18.2	25.3
Hemiplegia (%)	3.0	11.8
Renal disease (%)	19.5	25.6
Cancer (%)	21.1	18.3
AIDS/HIV (%)	1.3	1.7^*∗*^
Liver disease (%)	7.6	12.1
*Service groups (%)*		
ICU	0.3	0.6
Medicine	47.1	53.4
Neurology	14.4	23.5
OBGYN	2.4	0.7
Surgery	35.9	21.7

^*∗*^Variables that did not have a *p* value <0.05 compared to bCAM or CAM-ICU negative.

**Table 2:** Description of falls.

	Patient falls (*n* = 300)
Age (mean, SD)	57.9 ± 16.1
Age (median)	61.0
Female	128 (42.7%)
Unique patient falls	284
1 fall	270 (95.1%)
2 falls	12 (4.2%)
>2 falls	2 (0.7%)
Pre-fall Morse risk score documented	259 (86.3%)
Fall risk score (mean, SD)	68.9 ± 23.0
Fall risk score (median)	60.0
High fall risk (score >50)	193 (74.5%)
Injury sustained	47 (15.7%)

**Table 3:** Multivariable logistic regression adjusted odds ratio for falls.

	Odds ratio (OR)	95% lower OR	95% upper OR	*p* value
bCAM positive	2.809	2.125	3.696	0.0001
Age 65+ (ref. 18–64)	1.152	0.891	1.485	0.2780
Sex: male (ref. female)	0.702	0.545	0.900	0.0053
Marital status: married (ref. unmarried)	0.763	0.594	0.978	0.0328
Distance to MUSC > 50	1.181	0.925	1.509	0.1821
Poverty	1.237	0.962	1.582	0.0961
Anticholinergics	1.658	1.248	2.228	0.0004
Antipsychotic	1.815	1.416	2.328	0.0001
Opioid	1.316	0.897	1.992	0.1648
Benzo	2.033	1.562	2.658	0.0001
Dementia	0.733	0.395	1.249	0.2666
Alcohol abuse	1.370	0.927	1.987	0.1126
Congestive heart failure	1.238	0.919	1.650	0.1580
Cerebrovascular disease	1.458	1.020	2.050	0.0391
Peptic ulcer disease	2.283	1.234	3.881	0.0104
Cancer	1.368	1.149	1.033	0.0292
Liver disease	1.391	1.206	0.946	0.0915
*Service specialty: medicine (ref. medicine)*				
ICU	3.638	0.971	9.821	0.0545
Neurology	0.915	0.612	1.344	0.6556
OBGYN	2.824	1.475	5.036	0.0026
Surgery	1.134	0.846	1.516	0.3985

## Data Availability

The inpatient admission patient data used to support the findings of this study are restricted by the Institutional Review Board (IRB) of the Medical University of South Carolina in order to protect patient privacy. The data used to support the findings of this study are available from Benjamin Kalivas, kalivas@musc.edu for researchers who meet the criteria for access to confidential data.
